# Exercise-Induced Changes of Multimodal Interactions Within the Autonomic Nervous Network

**DOI:** 10.3389/fphys.2019.00240

**Published:** 2019-03-29

**Authors:** Solveig Vieluf, Tanuj Hasija, Rasmus Jakobsmeyer, Peter J. Schreier, Claus Reinsberger

**Affiliations:** ^1^Institute of Sports Medicine, University of Paderborn, Paderborn, Germany; ^2^Signal and System Theory Group, University of Paderborn, Paderborn, Germany

**Keywords:** canonical correlation analysis, multimodal approach, electrodermal activity, heart rate, temperature regulation, physical activity, autonomic nervous system

## Abstract

Physical exercise has been shown to modulate activity within the autonomic nervous system (ANS). Considering physical exercise as a holistic stimulus on the nervous system and specifically the ANS, uni- and multimodal analysis tools were applied to characterize centrally driven interactions and control of ANS functions. Nineteen young and physically active participants performed treadmill tests at individually determined moderate and high intensities. Continuous electrodermal activity (EDA), heart rate (HR), and skin temperature at wrist (Temp) were recorded by wireless multisensor devices (Empatica^®^ E4, Milan, Italy) before and 30 min after exercise. Artifact-free continuous 3 min intervals were analyzed. For unimodal analysis, mean values were calculated, for bimodal and multimodal analysis canonical correlation analysis (CCA) was performed. Unimodal results indicate that physical exercise affects ANS activity. More specifically, Temp increased due to physical activity (moderate intensity: from 34.15°C to 35.34°C and high intensity: from 34.11°C to 35.09°C). HR increased more for the high (from 60.76 bpm to 79.89 bpm) than for the moderate (from 64.81 bpm to 70.83 bpm) intensity. EDA was higher for the high (pre: 8.06 μS and post: 9.37 μS) than for the moderate (pre: 4.31 μS and post: 3.91 μS) intensity. Bimodal analyses revealed high variations in correlations before exercise. The overall correlation coefficient showed varying correlations in pretest measures for all modality pairs (EDA-HR, HR-Temp, Temp-EDA at moderate: 0.831, 0.998, 0.921 and high: 0.706, 0, 0.578). After exercising at moderate intensity coefficients changed little (0.828, 0.744, 0.994), but increased substantially for all modality pairs after exercising at high intensity (0.976, 0.898, 0.926). Multimodal analysis confirmed bimodal results. Exercise-induced changes in ANS activity can be found in multiple ANS modalities as well as in their interactions. Those changes are intensity-specific: with higher intensity the interactions increase. Canonical correlations between different ANS modalities may therefore offer a feasible approach to determine exercise induced modulations of ANS activity.

## Introduction

Complex functionality of the autonomic nervous system (ANS) is achieved by task-specific modulation of the organization of several organ-specific subnetworks as well as their interrelation (Jänig, 2006; Shaffer et al., 2014). Organ-specific regulative mechanisms of ANS, like heart-rate (HR) control or electrodermal activity (EDA), are integrated via a via a central autonomic network (CAN). The CAN consists of a network of cortical structures, such as left amygdala, right anterior and left posterior insula, and midcingulate cortices and subcortical structures (which form the “core” of the ANS control network), such as thalamus and brainstem (Beissner et al., 2013; Critchley and Harrison, 2013; Macey et al., 2016). ANS activity is therefore characterized by a finely coordinated interplay between activation, mediated by sympathetic modulation, and inhibition, mediated by parasympathetic modulation, for nearly all organs and body systems. Usually, i.e., within one organ-specific subsystem, sympathetic and parasympathetic activity are antagonistic but rely on intact central regulation and are subject to high day-to-day variations with poor systematics (Bellenger et al., 2017). More systematic central alterations of the CAN due to specific stressors like physical exercise may perturb organ functions (Tulppo et al., 2011) and thus alter ANS activity. Indeed, the interactions across several ANS subsystems are indicative of various ANS states (Bartsch et al., 2015; Schulz et al., 2016). Therefore, the analysis of the interrelation of subsystems of the ANS in addition to analyzing changes within each subsystem may provide additional insights into alteration of ANS control to physical exercise.

In the context of sports and exercise, the best described subsystem of ANS is the cardiac autonomic control. HR is a direct result of sympathetic and parasympathetic influences on the heart (Achten and Jeukendrup, 2003; Buchheit, 2014; Bellenger et al., 2017). Besides changes in the cardiac system, changes in the electrodermal and the thermoregulatory system have also been described. Due to its unique neurotransmitters (acetylcholine is used pre- and postganglionically) EDA represents sympathetic activity that is mediated by sympathetically stimulated eccrine sweat glands, which is linked to arousal and other stimuli (Boucsein, 2012). Moreover, skin temperature represents an additional easily obtainable parameter that is involved in thermoregulatory sympathetic processes in the ANS (Venables, 1991; Yoshihara et al., 2016). In general, regulation of ANS activity depends on a variety of factors (e.g., age and gender, psychology, circadian rhythm, etc.) resulting in high inter- and intraindividual variability (Al Haddad et al., 2011; Bellenger et al., 2017). As a consequence, ANS parameters have high sensitivity but poor specificity. Based on these findings, physical exercise might affect all subsystems of the ANS including their control, and therefore multimodal or multifaceted consideration of ANS subnetworks by assessing various organs and combining parasympathetic and sympathetic parameters may help generate additional insights into regulative processes in the CAN.

Besides the type of exercise, intensity is decisive for the strength of effects on regulative function in ANS, e.g., the balance between parasympathetic and sympathetic activity (Borresen and Lambert, 2008). For example, HR increases depend on exercise intensity (Seiler et al., 2007; Borresen and Lambert, 2008; Al Haddad et al., 2011). This effect is described during and right after exercise. However, sports and exercise-related effects also occur long-term, resembling adaptations to chronic stimuli. Focusing on the time scale of effects on ANS regulation during and in the context of exercise, a differentiation in acute (minutes to hours) and probably long-term effects (month and years following exercise) might be appropriate (Task Force of The European Society of Cardiology and The North American Society of Pacing and Electrophysiology, 1996). Changes within the cardiac autonomic balance depend on exercise intensity, while higher intensity was associated with post-exercise changes in autonomic balance indicating sympathetic predominance (Parekh and Lee, 2005; James et al., 2012; Esco et al., 2015). However, a combined analysis of exercise-induced effects in different ANS subsystems or modalities is rare. Boettger et al. (2010) reported changes of heart rate variability (HRV) and EDA in a study on incremental exercise levels and reported correlations between EDA and cardiac measures for low exercise intensities. Nevertheless, systematic approaches to analyze multimodal measures are still missing.

In this study, we aimed at detecting exercise-induced changes within different ANS subsystems and the change of their interactions after physical exercise. We selected HR, EDA, and skin temperature (Temp) as relevant measures of ANS subnetworks because physiological mechanisms as well as practical applications are well described within each subsystem. Also, all subnetworks are interrelated on different timescales. Further, they show a high practical relevance in the field of sports and exercise, and they are easy to measure. As physical activity induces sympathetic stress to the ANS, we assumed that for unimodal measures of EDA, HR, and Temp, the mean level increases in the acute setting. Further, we assumed that this effect becomes stronger with increasing intensity of the exercise. As cross-modal correlations might allow inferring CAN activity, we added analysis of multimodal interactions to unimodal analysis. For this multimodal analysis we expected that interrelations change from pre- to posttest. To analyze multimodal interactions among different ANS measures, we employed canonical correlation analysis (CCA) and multiset CCA (mCCA). These two techniques have been widely applied in numerous fields to study linear dependence among data sets (Hotelling, 1936; Thompson, 1984; Correa et al., 2010). However, the results obtained from these techniques are misleading when the number of observations (in this study, the number of participants) is small compared to the dimensions of the data sets (in this study, the number of time points in the recorded ANS time series) (Pezeshki et al., 2004). To overcome this challenge, we apply a technique specifically developed for this setting to reliably determine the number of correlated components and their correlation strengths among different ANS measures (Song et al., 2016).

## Materials and Methods

### Participants

Data of 5 min recordings during rest before and after exercise from 24 male students were recorded (see Table 1 for detailed characteristics of final sample). All participants were examined by an experienced sports physician (including resting and exercise ECG) and found healthy without limitation on physical exercise.

**Table 1:** Characteristics of participants included in the final sample.

	Age in years	Height in cm	Weight in kg	velocity at 60% VO_2_max	velocity at 95% VO_2_max	VO_2_max
Mean	24.16	183.68	79.18	9.54	15.11	48.84
SD	2.89	5.98	10.84	1.18	1.87	7.57

### Experimental Procedure

The study protocol was approved by the ethics committee of the Medical Board Westphalia-Lippe and Muenster University (2016-229-f-S; ANS-profile) and determined to be in agreement with the Declaration of Helsinki. Written informed consent was obtained from all participants prior to enrolment in the study.

**FIGURE 1:**
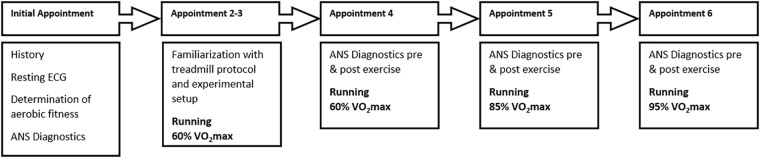
Visualization of the experimental procedure. Appointments were at least 48 h apart and took place at the same time for each participant.

Subjects participated in 6 treadmill tests (Figure 1). The initial appointment was used for the medical exam (including resting and exercise ECG) and determination of aerobic fitness VO_2_max (MetaLyzer 3B, Cortex Biophysik GmbH, Leipzig, Germany) by a step protocol (start at 6 km/h with an increase of 2 km/h every 3 min) on a treadmill (HP Cosmos Pulsar 3P, Nussdorf – Traunstein, Germany). Based on the results from this appointment individual running intensities for 60% (moderate), 85%, and 95% VO_2_max (high) were calculated. 60% VO_2_max is defined as the transition zone between aerobic and anaerobic energy supply, which corresponds to the first ventilatory threshold and therefore represents moderate intensity (Scharhag-Rosenberger and Schommer, 2013). Furthermore, the intensity zone between 50 and 60% VO_2_max is characterized by regulative influences switching from parasympathetic to sympathetic dominance (Tulppo et al., 1998). 85% VO_2_max corresponds to the second ventilatory threshold (respiratory compensation point) and is therefore defined as high intensity at steady state. Based on observations during the tests, we concluded that the 85% intensity was perceived and performed very differently by the participants. This was confirmed by different durations during the test, subjective rating of exhaustion (Borg scale) and results. Therefore, we present the results in the Supplementary Material only (see Supplementary Table 1).

Ninety-five percentage VO_2_max is defined as high demanding intensity close to maximum load but at least performable over a longer period of time (Scharhag-Rosenberger, 2010). Appointments two and three were used to familiarize the participants with the procedure of ANS diagnostics and treadmill running at an individual intensity at moderate speed.

In the following three appointments individual running at the three predefined intensities, in the same order of 60, 85, and then 95% for all participants, for a maximum of 20 min was performed. Prior to all appointments, a warm-up phase of 5 min was executed. At least 48 h of rest was required between appointments. To standardize for circadian rhythmic, we chose the same time of measurement for all appointments of an individual subject.

ANS signals were recorded by wireless multisensor devices (Empatica^®^ E4, Milan, Italy). The sensor was placed at the participants’ left wrists. Successive data series of HR (sampling rate 1 Hz), EDA (sampling rate 4 Hz), as well as Temp (sampling rate 4 Hz) at the wrist were acquired during 5 min in supine position prior to exercise and 30 min post exercise. We selected 30 min post exercise to account individual time needed for cool down, exudation, and taking a shower to minimize thermoregulatory adaptions. Based on personal conversation with our subjects all had a shower at pleasant water temperatures, nobody showered specifically hot or cold.

### Data Analysis

Data analysis was performed using Matlab (R2017a, The MathWorks Inc., Natick, Massachusetts, United States). From each measurement a time window of three continuous minutes were manually selected to control for data quality and avoid movement artifacts. Based on data quality, 5 participants were excluded from further analysis, either due to loss of sensor connection, an incomplete HR recording, or lack of a movement-free segment. HR data was derived from the standardized manufacture software and was based on blood volume pulse (BVP) data and inter-beat-interval estimation (Garbarino et al., 2014). Raw data of the EDA signal were detrended, low-pass-filtered (butterworth filter 4th order with cut of frequency of 0.4 Hz) and smoothed (by using a moving average filter with the factor 9). Rejection of artifacts was performed manually for EDA. The rejected part was replaced by cubic, spline, or linear interpolation based on visual inspection according to the development of the EDA signal. Temp data was smoothed (factor 4).

For the unimodal analysis, mean Temp, mean EDA, and mean HR were calculated for each pre- and post-exercise segment. Statistical analysis was performed with SPSS (IBM, Version 22). Pre-Post (2; pre, post) × Intensity (2; moderate, high) repeated measures ANOVAs were conducted. Effect sizes were reported as partial Eta squares (η_p_^2^). Whenever sphericity was violated, Greenhouse-Geisser correction was applied. The level of significance was set at 5%. If sphericity was violated, Greenhouse-Geisser corrected *p*-values were reported. Significant effects were followed by Bonferroni-corrected pairwise comparisons.

CCA and mCCA were used to analyze bimodal and multimodal interactions between EDA, HR, and Temp data sets (Hotelling, 1936; Kettenring, 1971). The data sets for each modality were generated such that the recorded times series from each subject forms a column of the data matrix. Thus, the size of the data matrix for each modality is the number of time points times the number of participants.

Canonical correlation analysis linearly transforms the data sets and determines the so-called canonical correlations that measure the strength of linear association between the two data sets. These canonical correlations are normalized between 0 and 1 and ranked in decreasing order of their values. A canonical correlation equal to 1 indicates that a component in a data set is perfectly correlated with another component in the other data set, whereas a canonical correlation of 0 indicates an uncorrelated component. Therefore, the number of nonzero canonical correlations, *r*, represents the dimension of the correlated subspace or simply the number of components correlated between the two data sets. However, for this work, not only the number of correlated components but also their strength of correlation is interesting. This can be measured with an overall correlation coefficient, *ρ_c_* (Schreier and Scharf, 2010), which can be computed as a function of the nonzero *r* canonical correlations as

(1) $$\rho_{c}=1-\prod_{i=1}^{r}(1-k_{i}^{2}),$$

where *k_i_* denotes the *i*th canonical correlation. This overall correlation coefficient also relates to the mutual information between the two data sets if the two data sets were Gaussian distributed. Thus, high values of *ρ_c_* may be interpreted to mean that the two data sets share more information.

The canonical correlations can be computed in closed form using the auto- and cross-covariance matrices of the data sets (Scharf and Mullis, 2000). However, in practice, the covariance matrices are unknown and must be estimated from samples (in our case, each subject is regarded as one sample). If the number of samples is not large compared to the dimensions of the data (in our case, this corresponds to the number of time points in the recorded time series), the canonical correlations are significantly overestimated, i.e., their estimated values are much higher than the true values. In this work, the number of samples (subjects) is much smaller than the dimensions of the data sets (number of time points). In this case, many estimated canonical correlations are equal to 1, irrespective of the true number of correlated components, and all of them are nonzero (Pezeshki et al., 2004). This is the main challenge when applying CCA with a small number of samples.

A common solution to address this problem is to use a dimension-reduction preprocessing step [typically principal component analysis (PCA)], applied to each data set individually. The complication, however, is that PCA is designed to extract components that account for most of the variance within one data set, but these components are not necessarily the ones that account for most of the correlation between two data sets. Therefore, the number of dimensions in each data set to be kept by PCA needs to be determined jointly with the number of nonzero canonical correlations. To accomplish this, Song et al. (2016) presented three related techniques, which are adaptations of the classical Bartlett-Lawley test (Bartlett, 1941; Lawley, 1959) to the case of small number of samples. In this work, the hypothesis-test-based detector (Detector 1) from Song et al. (2016) is applied for the bimodal analysis of the physiological data. These techniques have also been successfully applied in biomedicine for fusing brain imaging data from different modalities (Levin-Schwartz et al., 2016). This approach is based on a sequence of binary hypothesis tests, and the level of significance for each test was set to 5%. It could be hypothesized that the higher the number of correlated components between modalities, the more complex their interaction is. If the interaction is limited to a linear relationship in a single dimension, this should indicate a rather simple interaction. On the other hand, multiple correlated components would indicate a more complex type of relationship between modalities.

There are several different ways of extending CCA to multiple data sets in order to investigate the interactions jointly across all three modalities, summarized in Kettenring (1971). All of these extensions fall under the common term mCCA. The idea is to extract one component from each data set such that these are maximally correlated with each other. The extracted components are commonly called source components (SCs). Subsequent components are extracted in a same way except that these are to be uncorrelated with previously extracted components. It is shown in Li et al. (2009) that mCCA enables blind source separation for multiple data sets.

Kettenring (1971) presented five different versions, for different cost functions, to perform mCCA. Thus, unlike CCA, mCCA is not unique and depends on the chosen cost function. However, all five versions reduce to CCA when the number of data sets is two. In this work, the generalized variance (GENVAR) cost function is used to perform mCCA. For GENVAR, the SCs are extracted such that they minimize the determinant of the SC correlation matrix. The SC correlation matrix, R, is a square matrix of dimension equal to the number of data sets and measures how the SCs are correlated with each other. For instance, the absolute value of the *ij*th element of R is the correlation coefficient between the extracted components of the *i*th and *j*th data sets, which is normalized between 0 and 1. The GENVAR mCCA has been widely applied in biomedicine, for instance in analyzing functional MRI data (Adali et al., 2015) and for fusing functional MRI, EEG, and structural MRI data (Levin-Schwartz et al., 2016).

However, mCCA suffers from the same problems as CCA when the number of samples is small compared to the dimensions of the data sets. The correlation coefficients for the extracted components are significantly overestimated and do not reflect the true correlation structure among these components (Asendorf and Nadakuditi, 2015). One possible solution is to again use a dimension-reducing pre-processing step (such as PCA) for each data set individually. But as with CCA, the components extracted from a PCA pre-processing step do not necessarily correspond to the components correlated across multiple data sets. There is no technique yet that jointly determines the required PCA dimensions and simultaneously performs mCCA. Our proposed solution is to estimate the PCA dimensions returned by the joint PCA-CCA detector of Song et al. (2016) for each pair of data sets and to choose the maximum dimension for each data set. This approach is based on the fact that the components correlated across all data sets are also correlated across a given pair of data sets. Hence, these components are retained in the dimension-reduced data sets using the PCA rank estimated by the joint PCA-CCA detector.

## Results

### Performance Diagnostics

Average maximum oxygen intake (VO_2_max) was 49.80 ± 7.65 ml/min/kg (see Table 1), which can be considered as moderate to high fitness level for this age group (Edvardsen et al., 2013). Average running speed was 9.7 ± 1.19 km/h for 60% and 15.4 ± 1.88 km/h for 95% VO_2_max (see Table 1). The treadmill running lasted 20 min at maximum, but was significantly shorter (mean time = 6 min 12 s) at 95% VO_2_max.

### Unimodal Results

**FIGURE 2:**
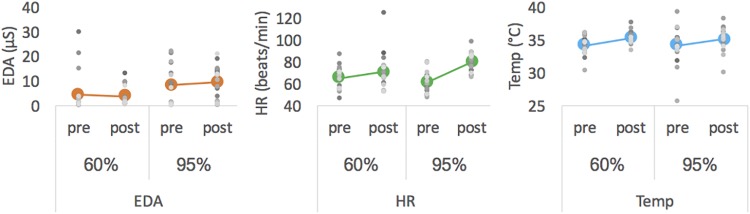
Mean EDA (left), mean HR (middle) and mean Temp (right) averaged for pre- and post-tests of each intensity. Colored thick marker represents group mean and each gray dot represents one participant.

Descriptive data are presented in Figure 2 and Table 2. Statistical analysis revealed a main effect of intensity for mean EDA [*F*(1,18) = 7.913, *p* = 0.003, η_p_^2^ = 0.305] with EDA level being lower at moderate than at high intensity. For HR measures, we obtained a significant interaction effect [*F*(1,18) = 8.278, *p* = 0.010, η_p_^2^ = 0.315]. Follow-up pairwise comparisons revealed a significant difference between 60% and 95% in the posttest (*p* = 0.026), with HR being higher after exercising at high than at moderate intensity, but not for pretest measures (*p* = 0.188). Pre- and post-test measures differed for 60% (*p* = 0.085) and 95% (*p* < 0.001). The latter showed a significant main effect of pre-post-test for HR [F(1,18) = 36.240, *p* < 0.001, η_p_^2^ = 0.668]. For Temp the main effects pre-post-test [*F*(1,18) = 15.374, *p* = 0.001, η_p_^2^ = 0.461] was significant, with Temp being higher for post- than for pre-test. All other effects failed to reach significance.

**Table 2:** Mean and SD of HR (bpm), mean EDA (μS) and Temp (°C) are presented per group.

Modality	Intensity	Pre		Post	
	**(% VO_2max_)**	**Mean**	***SD***	**Mean**	***SD***
HR	60	64.81	10.31	70.83	16.29
HR	95	60.76	10.69	79.89	8.52
EDA	60	4.31	8.30	3.66	3.91
EDA	95	8.06	8.40	9.37	6.51
Temp	60	34.15	1.48	35.34	0.90
Temp	95	34.11	2.78	35.09	1.81

### Bimodal Results

**FIGURE 3:**
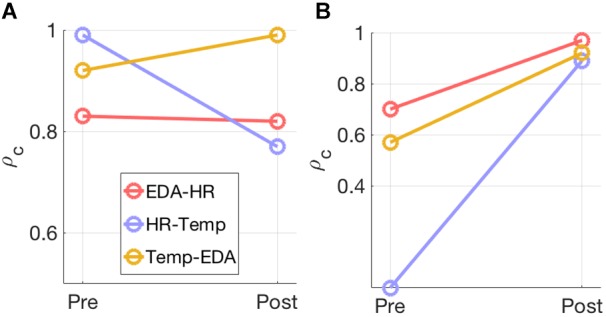
The overall correlation coefficient, ρ_c_, estimated for each modality pair at **(A)** 60% exercise intensity and **(B)** 95% exercise intensity is illustrated

Model selection using the joint PCA-CCA approach of Song et al. (2016) revealed significant correlated components in all measures for most modality pairs (Table 3). The maximum PCA rank was set to seven in accordance with the rules of Song et al. (2016). In the pre-test the number of correlated components differs between modality pairs and intensities. For the post-60% intensity, HR shows one correlated component with EDA and Temp each, while EDA and Temp show two components. In the 95% intensity condition, the number of components increases to two for all modality pairs and the canonical correlations increase for all components. This is summarized in Figure 3, which shows how the overall correlation coefficient varies from pre to post-exercise. The overall correlation coefficient, ρ_c_ is computed from the canonical correlations in Table 3 for each modality pair and at both moderate and high intensity. At moderate intensity, exercise does not seem to have a clear and specific effect on ρ_c_. However, at high intensity, there is a substantial and clear increase in ρ_c_ from pre- to post-test for all three modality pairs.

**Table 3:** Number of significant components and their strength of correlation are presented for each modality pair during pre- and post-test measures at moderate and high intensity.

		Pre-test		Post-test	
Modality pair	Exercise intensity	Number of significant components	Canonical correlations, (*k*_i_)	Number of significant components	Canonical correlations, (*k*_i_)
EDA-HR	60%	2	0.8, 0.73	1	0.91
HR-Temp		3	0.97, 0.93, 0.84	1	0.88
Temp-EDA		1	0.96	2	0.97, 0.95
EDA-HR	95%	1	0.84	2	0.94, 0.89
HR-Temp		0	0	2	0.86, 0.78
Temp-EDA		1	0.76	2	0.88, 0.82

### Multimodal Results

**FIGURE 4:**
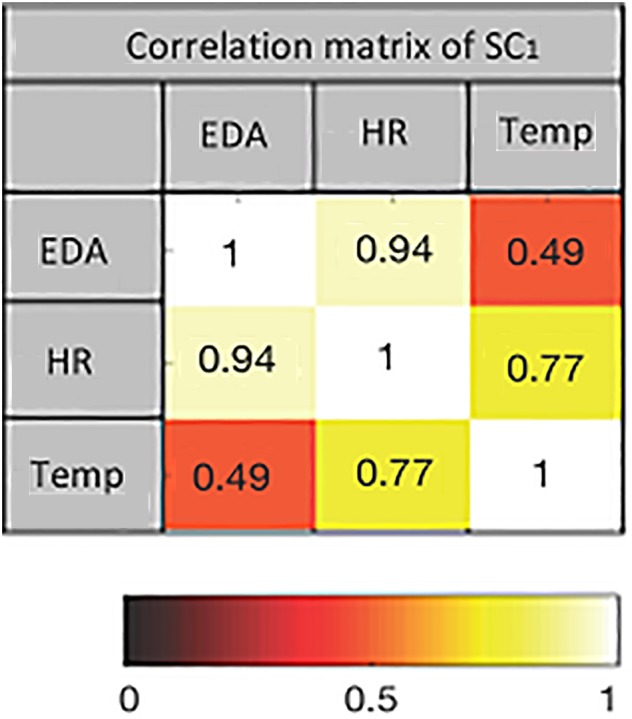
The heat map of the correlation matrix of first source component (SC) for pre-exercise data at 60% intensity. Color coding indicates the strength of correlation between pairs of modalities.

The first two SCs from pre- and post-test data for both intensities are extracted using mCCA. The dimension of each data set after applying PCA is equal to seven. This is the maximum PCA rank estimated by the PCA-CCA detector for each pair of data sets. The correlation matrices of the SCs show the joint interactions among the extracted components. An example of the correlation matrix of the first SC from pre-exercise data at 60% intensity is shown in Figure 4. The values indicate how strongly the components jointly extracted from the three modalities are correlated. The corresponding heat map is also shown.

**FIGURE 5:**
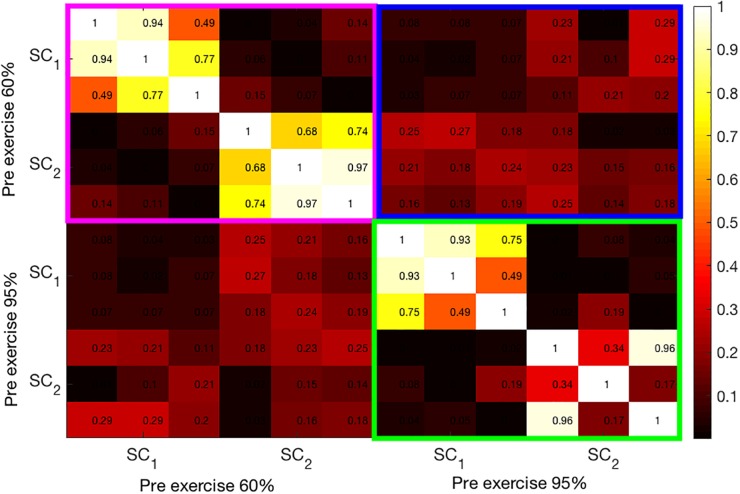
Illustration of the correlation structure within pre-test measures. On the x and y axis the first 2 source components (SC) are depicted. Highlights indicate the correlation of the maximally correlated source components within (pink and green square) and between intensities (blue square).

In Figure 5, a heat map of correlation coefficients between the first two SCs from pre-exercise data is shown. The magenta-framed rectangle contains the correlations among the SCs at 60% intensity. The 3 x 3 off-diagonal blocks within the magenta rectangle are almost zero as the second set of components (SC2) is extracted under the constraint that they be uncorrelated with the first set (SC1). Similarly, the green-framed rectangle displays the correlations among the two SCs extracted at 95% intensity. In line with bimodal results, the correlations among the pre-exercise components at both intensities are generally not very high and quite variable across the modalities. Finally, the blue-colored rectangle shows the correlation coefficients among the components at 60% and 95% intensities. These components are almost uncorrelated.

**FIGURE 6:**
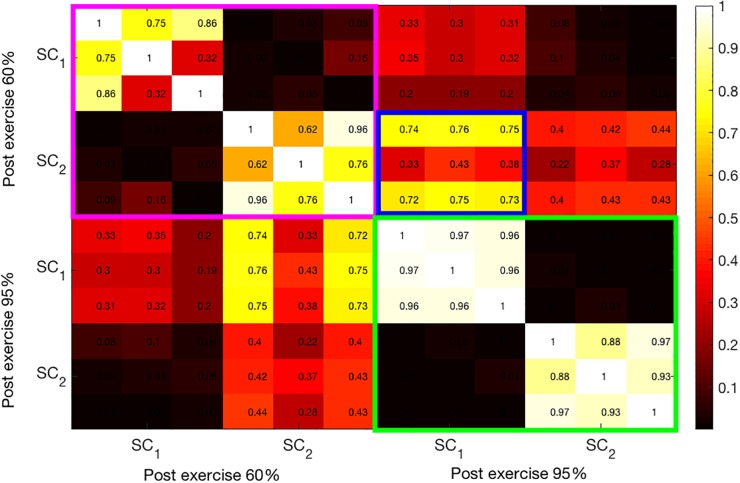
Illustration of the correlation structure within post-test measures. On the x and y axis the first 2 source components (SC) are depicted. Highlights indicate the correlation of the maximally correlated source components within (pink and green square) and between intensities (blue square).

Similarly, Figure 6 shows the correlation coefficients between the extracted components from the post-exercise data. As in the bimodal results, the correlations between the SCs at 95% intensity are high. Moreover, these components have high correlation coefficients across all three modalities, as opposed to pre-95% intensity data, where the components (green-colored rectangle in Figure 5) have high correlations only among one or two pairs of modalities. Finally, the first and the second SCs at 60 and 95% intensities, respectively, have higher correlation coefficients (average 0.6) than pre-exercise SCs, indicating that these are related components. This can be seen in the blue-framed rectangle.

## Discussion

The aim of the current study was to elucidate changes within and between ANS subsystems related to physical activity. Considering the ANS as a complex network whose functionality is affected by the situation-specific adaptation of the interplay of several subsystems (Jänig and Häbler, 2000; Shaffer et al., 2014; Bartsch et al., 2015; Schulz et al., 2016), we aimed to illustrate how physical exercise, as a stimulus leading to central ANS modulations, alters these interactions. The resting state of ANS activity depends on the central processing of a variety of afferently transmitted information to the central network of the ANS. If a strong afferent stimulus alters this central regulation, efferent control of ANS subsystems are altered. To gain insights into those exercise-induced changes, we analyzed time series of HR, EDA, and Temp data during rest pre and post exercise in each modality, and we proposed analysis tools to reveal bimodal and multimodal interactions and presented the results. Unimodal results indicate that physical exercise affects the ANS. Bimodal analysis showed high variations in pre-test correlations, while in post-test the correlations were higher especially after high-intensity exercise. We used mCCA to infer ANS state changes based on signal interactions across all modalities. Our results indicate that in measures taken before the exercise, cross-modality interactions exist but do not seem to follow a specific pattern, while in post-exercise measures the cross-modality interactions increase and show similarities between the different intensity tests, indicating an exercise-specific organization of the ANS modalities EDA, HR, and Temp.

### Physical Activity as a Holistic Stimulus Affects the Autonomic Network

In this study we confirmed that physical exercise has an impact on several subsystems of the ANS. The previously described sympathetic activation due to physical exercise (Parekh and Lee, 2005; James et al., 2012; Esco et al., 2015) most likely affects all ANS modalities reported in this study depending on the intensity. More precisely, from pre- to post-test Temp and HR increase (for similar results for HR see: Seiler et al., 2007; Borresen and Lambert, 2008; Al Haddad et al., 2011), the latter more so with higher intensity. Furthermore, the HR differs between intensities only after and not before exercise, indicating similar cardiac activity levels before exercising, which could be expected as the time of measurement was kept constant for each subject. EDA level is higher with higher intensity. However, large intra- and inter-individual variability might mask these effects. Especially pre-test measures underlie a high day-to-day variability as previously described (Al Haddad et al., 2011; Bellenger et al., 2017). In general, the variability within the group is reduced after exercise, which might indicate that the ANS of different individuals reorganizes in a similar way to exercising, which decreases the influence of day-to-day variability. In addition to analyzing the changes within each subsystem, we used CCA to infer the centrally moderated changes in the interaction of subsystems.

Bimodal interactions were tested via PCA-CCA analysis. Analysis confirms the day-to-day variability in a sense that the correlation structures differ strongly in pre-test measures. In post-test measures the number of correlated components is similar across modality pairs. However, the strength of correlation differs between modality pairs. Especially EDA and Temp show strong correlations (see also Figure 6), which might be the result of common or similar anatomical and functional pathways and the contribution to thermoregulation. However, similar correlations between HR and EDA, as well as HR and Temp indicate that post-test measures cannot solely be due to thermoregulatory effects. The results also show intensity effects. In the post-test the number of correlated components and their strengths are higher for the high than for the moderate intensity. For the high intensity, all pairwise correlations show an increase from pre- to post-test, while the picture is mixed for the moderate intensity. Moderate intensities may have a more regenerative effect on the ANS, so that homeostasis is reestablished, and physical stress might already be diminished 30 min after exercise. On the other hand, the high intensity presents a stronger effect that lasts longer, and therefore sympathetic activation, especially indicated by increases in EDA, is prominent in our post-test measures. Subsystems might still underlie a joint central control that contributes to reestablishing homeostasis and balance within the ANS. In line with the bimodal results and based on the idea of a jointly centrally interconnected regulation of ANS subsystems, a multimodal approach may offer an approach to characterize the central integration of multiple modalities. On a descriptive level, components with correlations across three modalities, with average correlations higher than 0.8, can be detected. Detected components are higher in the post- than in the pre-test measures. Especially in the high-intensity condition the post-test correlations increase strongly and are higher than for the moderate intensity, providing a first hint toward the assumed integration of subsystems in a CAN (Beissner et al., 2013; Critchley and Harrison, 2013; Macey et al., 2016). The finding that the post-test components correlate across intensities offers an interesting starting point for further investigations to describe exercise-specific organizations of the ANS.

In a broader sense, our results resemble findings in other domains. Changes in ANS states have been frequently described for psychological stress (for an overview see Oken et al., 2015). Looking at the dynamic responses of the ANS to a stressor shows that it is pushed away from a baseline state and may shift to a less efficient attractor state, i.e., a specific network organization related to the physiological response of the ANS to the stressor (Oken et al., 2015). Returning to the original state is related to an allostatic load that depends on the strength of the stressor and the system’s ability to recover (Oken et al., 2015). Besides psychological stress we showed that physiological stress induces specific changes within the ANS that alters the organization within and between ANS subnetworks (Borresen and Lambert, 2008; Stanley et al., 2013; Bär et al., 2016). However, the method we propose in this paper to jointly analyze ANS signals was so far used in an explorative way and therefore offers several possibilities for modifications and expansions.

### PCA-CCA and mCCA to Access Multimodal Interactions

A joint PCA-CCA technique (Song et al., 2016) was employed for bimodal analysis of different ANS measures. The usefulness of this technique is that it not only provides the degree of similarity between two different measures but also detects the number of correlated components between them. This is essential especially in this study as the number of participants is small compared to the number of time points in each ANS measure.

Both CCA and mCCA are designed to extract components that are highly correlated, i.e., explain most of the linear dependence among two or more data sets. While CCA has a closed form and a unique solution, it is limited to two data sets. For more than two data sets, the components extracted using CCA from different pairs of data sets have to be related to each other to analyze their correlation across all the data sets. This can be difficult, especially in case of small sample support. mCCA extends CCA by jointly analyzing all the data sets. However, mCCA in small sample support has not yet received the attention it deserves. One of the challenges is to apply a pre-processing step like PCA to reduce the dimensions of all the data sets such that all the correlated components for the mCCA step are retained. Furthermore, testing the correlation structure of the extracted components from mCCA remains a challenge. For example, for three data sets, a SC could be highly correlated across only a pair of data sets and uncorrelated with the third set. There could be another component moderately correlated across all the data sets. Therefore, detecting the number of components along with their correlation structure for a small number of samples still remains an open problem in the literature. In this context, it needs to be emphasized that so far, there exists no technique to determine which of the correlations between components (e.g., as shown in Figure 5, 6) are statistically significant. While the PCA-CCA detector in Song et al. (2016) detects the number of correlated components, the mCCA approach used in this work only computes correlation coefficients between components but does not estimate the number of correlated components.

### Limitations and Outlook

Our study has several shortcomings. Based on the multimodal device we obtained data with comparably low sampling rates. On the other hand, it offers a precise synchronization of the different time series, which is beneficial for joint analysis. Also, by choosing one single device for the measurement we were limited in modality selection. Future studies might add respiratory rate or blood pressure changes over time to the analysis. A central source of the described ANS changes can only be assumed based on ANS anatomy and physiology, since interactions within ANS subsystems are modulated by a central network (Jänig, 2006). Proofing the central origin of mCCA changes in peripheral ANS channels might be methodologically challenging, but will be of interest for future studies. Based on our pre-post-test design, we cannot illustrate the evolution of the signals or their interrelation. Measuring the post-test 30 min after exercising allows participants to cool down and exudate, but it offers insight only into a very specific window of regulative procedures related to exercise. We have not controlled individual strategies of recovery after exercise. Neither water temperature, nor body position for relaxation nor clothing was standardized in the 30 min after exercise. Future studies should control this phase more precisely and systematically control laboratory settings like temperature or humidity. Since the experiments were very time-consuming, we included only 24 participants, but a bigger sample size would lead to more reliable results. If a bigger cohort is difficult to recruit, one might consider analyzing changes in the ANS in relation to stronger stimuli, like psychological stress or neurological diseases such as epilepsy with a focus on seizure-induced changes, which are all known to alter the ANS. A longer intervention might also alter the organization within the ANS. However, other intensities would have to be used as our participants could not maintain the high intensity for 20 min. Different test durations at different intensities might also impact our current data. Additionally, it would be of great interest to test each intensity several times to also see if the variability between days differs between intensities with the aim to establish baseline measures. Also, the evaluation of ANS interactions at other respiratory and metabolic thresholds might be of interest in the future. Another limitation of our study is the selection of modalities. We selected HR, EDA, and Temp based on their physiological meaning of regulation of ANS activity and the availability of continuous measures. This might be of interest in future studies in order to better characterize the interplay of the sympathetic and parasympathetic branches within multiple organ systems.

## Conclusion

In sum, the current study confirmed that subsystems of the ANS exchange information at all times. These interactions presumably allow flexible adaptation to different situations, but they result in high pre-test variability. Therefore, unimodal approaches might underestimate effects. We propose a multimodal analysis based on CCA to gain additional insights into cross-modality changes. Our results suggest that physical activity seems to be a holistic stimulus that alters the overall interrelation of the subsystems. The effect of physical exercise depends on the intensity and seems to have different effects on altering the regulative functioning. This might be of future interest for training control as the monitoring can provide information on what kind of intensity is best to achieve a certain ANS state, e.g., for the last training before a race or match. Based on these stimulus-specific changes we conclude that the signals’ correlation structure might be indicative for a stimulus specific organization of the ANS. Further studies should be performed with bigger data sets and other stimuli.

## Author Contributions

SV, TH, RJ, PS, and CR contributed to the conception of the work, drafted parts of the manuscript, approved the final version, and agreed to be accountable for the complete work.

## Conflict of Interest Statement

The authors declare that the research was conducted in the absence of any commercial or financial relationships that could be construed as a potential conflict of interest.

## Abbreviations

ANS: autonomic nervous system
BVP: blood volume pulse
CAN: central autonomic network
CCA: canonical correlation analysis
ECG: electrocardiogram
EDA: electrodermal activity
HR: heart rate
mCCA: multiset CCA
PCA: principal component analysis
SC: source component
Temp: skin temperature at wrist
VO_2_max: maximal oxygen consumption.
