# Supplementary material for: Exercise-Induced Changes of Multimodal Interactions Within the Autonomic Nervous Network
Source: Front Physiol. 2019 Mar 29;10:240. doi: 10.3389/fphys.2019.00240 (PMC6449462; doi:10.3389/fphys.2019.00240)
Supplement: Supplementary file 1 [file Table_1.DOCX]

Supplementary material

**Table 1** Uni and bimodal results for 85% intensity.

|  | Pretest | | | Posttest | |
| --- | --- | --- | --- | --- | --- |
| Modality | mean | SD | mean | | SD |
| HR | 60.77 | 9.74 | 80.14 | | 8.43 |
| EDA | 8.08 | 11.67 | 9.84 | | 6.60 |
| Temp | 34.88 | 1.97 | 36.18 | | 1.15 |
| Modality pair | Number of significant components | Canonical correlation coefficients | Number of significant components | | Canonical correlation coefficients |
| EDA-HR | 1 | 0,97 | 1 | | 0,46 |
| HR-Temp | 1 | 0.52 | 0 | | 0 |
| Temp-EDA | 2 | 0.98, 0.9 | 2 | | 0.97, 0.94 |
